# Effects of G and SH Truncation on the Replication, Virulence, and Immunogenicity of Avian Metapneumovirus

**DOI:** 10.3390/vaccines12010106

**Published:** 2024-01-21

**Authors:** Seung-Min Hong, Eun-Jin Ha, Ho-Won Kim, Seung-Ji Kim, Sun-Min Ahn, Se-Hee An, Gun Kim, Suji Kim, Hyuk-Joon Kwon, Kang-Seuk Choi

**Affiliations:** 1 Laboratory of Avian Diseases, Department of Farm Animal Medicine, College of Veterinary Medicine and BK21 PLUS for Veterinary Science, Seoul National University, Seoul 088026, Republic of Korea; topkin@snu.ac.kr (S.-M.H.); flower678@snu.ac.kr (E.-J.H.); iamkhw52@snu.ac.kr (H.-W.K.); seungji910@snu.ac.kr (S.-J.K.); vicky.ahn@snu.ac.kr (S.-M.A.); 2 Research Institute for Veterinary Science, College of Veterinary Medicine, Seoul 08826, Republic of Korea; kwonhj01@snu.ac.kr; 3 Animal and Plant Quarantine Agency, Gimcheon 39660, Republic of Korea; ashpri@korea.kr; 4 Laboratory of Veterinary Pharmacology, Research Institute of Veterinary Science, College of Veterinary Medicine, Seoul National University, Seoul 088026, Republic of Korea; smilesssss@snu.ac.kr; 5 Department of Nuclear Medicine, Seoul National University Bundang Hospital, Seongnam 13620, Republic of Korea; sujeeksj@snu.ac.kr; 6 Laboratory of Poultry Medicine, Department of Farm Animal Medicine, College of Veterinary Medicine and BK21 PLUS for Veterinary Science, Seoul National University, Seoul 088026, Republic of Korea; 7 Institutes of Green-bio Science Technology (GBST), Farm Animal Clinical Training and Research Center (FACTRC), Seoul National University, Pyeongchang 25354, Republic of Korea; 8 GeNiner Inc., Seoul 08826, Republic of Korea

**Keywords:** avian metapneumovirus, G and SH truncation, virus replication, attenuation, immunogenicity

## Abstract

Four mutants varying the length of the G and SH genes, including a G-truncated mutant (ΔG) and three G/SH-truncated mutants (ΔSH/G-1, ΔSH/G-2, and ΔSH/G-3), were generated via serially passaging the avian metapneumovirus strain SNU21004 into the cell lines Vero E6 and DF-1 and into embryonated chicken eggs. The mutant ΔG particles resembled parental virus particles except for the variance in the density of their surface projections. G and G/SH truncation significantly affected the viral replication in chickens’ tracheal ring culture and in infected chickens but not in the Vero E6 cells. In experimentally infected chickens, mutant ΔG resulted in the restriction of viral replication and the attenuation of the virulence. The mutants ΔG and ΔSH/G-1 upregulated three interleukins (IL-6, IL-12, and IL-18) and three interferons (IFNα, IFNβ, and IFNγ) in infected chickens. In addition, the expression levels of innate immunity-related genes such as *Mda5*, *Rig-*I, and *Lgp2*, in BALB/c mice were also upregulated when compared to the parental virus. Immunologically, the mutant ΔG induced a strong, delayed humoral immune response, while the mutant ΔSH/G-1 induced no humoral immune response. Our findings indicate the potential of the mutant ΔG but not the mutant ΔSH/G-1 as a live attenuated vaccine candidate.

## 1. Introduction

Avian metapneumovirus (aMPV) is one of the major respiratory viruses that have a significant economic impact on poultry worldwide. It causes acute, highly contagious upper respiratory tract infections and is also associated with swollen head syndrome (SHS) in chickens [1]. aMPV is an RNA virus that belongs to the *Metapneumovirus* genus in the family *Pneumoviridae* [2]. Another member of the *Metapneumovirus* genus is the human metapneumovirus (hMPV), a major respiratory pathogen in children. There are currently four distinct subtypes of aMPV (A, B, C, and D) with genetic and antigenic differences. Subtypes A and B are the major subtypes causing significant economic losses in chickens, and they are prevalent in many countries in Europe, Asia, and Africa [3,4,5]. Subtype C causes acute respiratory infections in the turkey industry in North America (especially in the United States) and has also been reported in farmed ducks (in France), pheasants (in South Korea), and Muscovy ducks and chickens (in China), as well as some wild birds (in the United States) [6,7,8,9,10]. Subtype D has been reported in only one case in turkeys in France in 1985 [11].

The viral genome is in the form of a non-segmented, single-stranded, negative-sense RNA, and it contains eight genes (3′-N-P-M-F-M2-SH-G-L-5′) that encode nine proteins, including nucleoprotein (N), phosphoprotein (P), matrix protein (M), fusion protein (F), second matrix protein (M2-1 and M2-2), small hydrophobic protein (SH), attachment glycoprotein (G), and large RNA-dependent RNA polymerase (L) [12,13,14], three internal structural proteins (N, P, and L), and viral RNA molecules form the ribonucleoprotein (vRNP) complex, which is required for transcription and replication [12]. There are three external structural glycoproteins, G, F, and SH, located on the envelope, and they are responsible for the attachment and entry of the virus into susceptible cells. Among them, the G glycoprotein is known to be the viral attachment protein. However, it has been reported that F can induce virus–cell fusion by binding to cell receptors without interacting with the viral G protein in both in vivo and in vitro studies with hMPV [15]. The G gene of aMPV isolates exhibits extensive genetic variations, owing to its nature, such as point mutation, insertion, and deletion, which occur during virus evolution. In addition, recent studies have shown that the G gene length variation resulted either from a truncation of the G gene during serial passages in Vero cells or during continuous circulation in the field [16,17]. They were found to be replication-competent in vitro despite the impairment of the G glycoprotein [17,18]. For that reason, the role of aMPV G in its relationship with virus replication, pathogenicity, and immunogenicity has caused a great deal of scientific curiosity.

In the present study, we generated various mutants with different forms of G and/or SH truncation during the serial passage of field isolates of aMPV suspected of infection in various viral culture systems. The genetic and biological properties of the resulting mutants were characterized via a comparison with the parental virus. In addition, the virulent and immunogenic properties of the mutant viruses were determined in vivo in experimental models of specific pathogen-free chicken and mice.

## 2. Materials and Methods

### 2.1. Virus and Cells

The aMPV strain SNU21004, belonging to subtype B of aMPV, was used in this study. The SNU21004 virus was isolated from the nasal cavity of a dead bird (37 days old) with respiratory diseases on a layer chicken farm in Korea in 2021 and propagated in a chicken embryo tracheal organ culture (TOC) with a 1:1 mixture of Medium-199 and Ham F10 in 15 mL culture tubes in a 37 °C shaking incubator, as previously described [19,20]. The propagated virus was finally passaged eight times in TOC and finally stored at −80 °C until use. Two cell lines, including African-green-monkey-derived Vero E6 cells (ATCC no. CRL-1586) and chicken-fibroblast-derived DF-1 cells (ATCC No. CRL-12203), were used. Both cell lines were maintained in Dulbecco’s Modified Eagle Medium (DMEM, Gibco, Waltham, MA, USA) supplemented with 10% (*v*/*v*) fetal bovine serum (Gibco, Waltham, MA, USA) and a 1% (*v*/*v*) antibiotic–antimycotic solution (Gibco, Waltham, MA, USA). Specific-pathogen-free (SPF) embryonated chicken eggs (ECEs; Valo Biomedia, Adel, IA, USA) were also used.

### 2.2. Generation of aMPV Mutants with Different Genome Lengths

**Figure 1:**
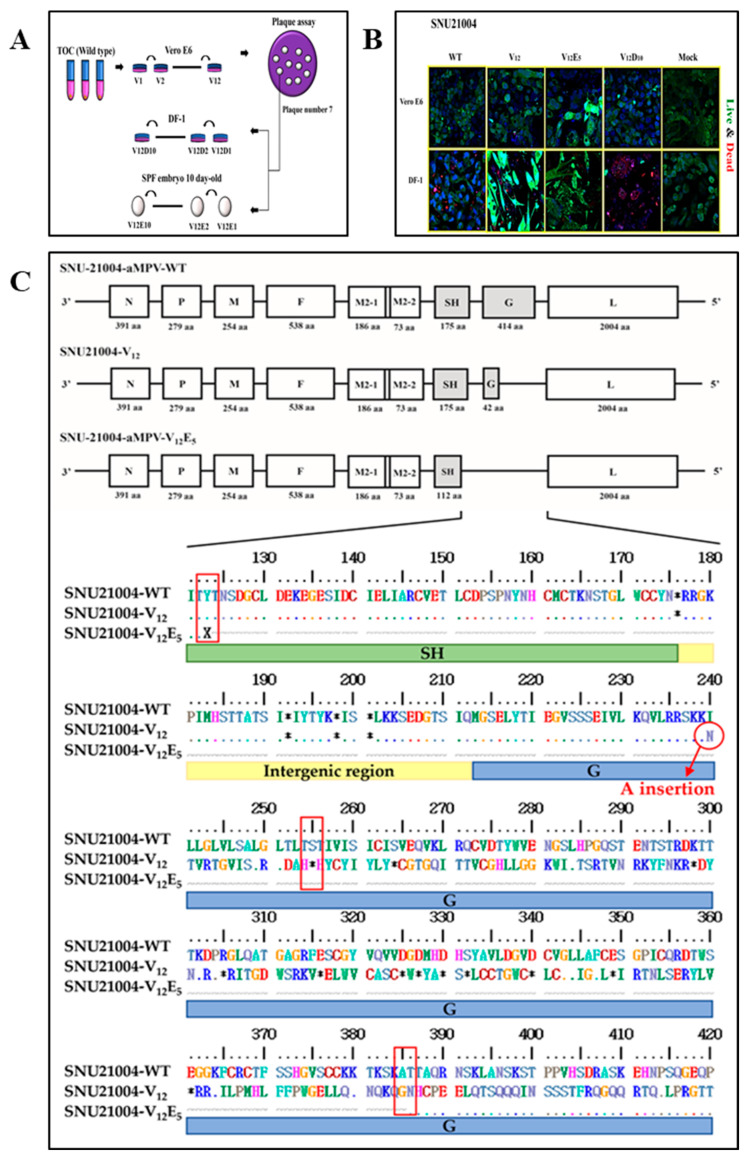
Schematic representation of the genomic architecture of the wild-type and variants of SNU21004, including a comparison with the reference genome. (**A**) Schematic diagram of the cell adaptation process. (**B**) Analysis of morphological alterations in cells subjected to cytopathic effects following infection with a cell-adapted virus. Co-staining with three colors (FITC, H3342, and Texas Red) was performed using cell-permeable and impermeable dyes to distinguish live (green) and dead (red) cells. (**C**) The occurrence of early stop codon (*) formation in SNU21004-V_12_ and the large deletion in the SH-G region of the V_12_E_5_ are highlighted with red boxes in the amino acid sequences.

aMPV mutants with different-length truncations of aMPV G and/or SH were generated in vitro through serial passages of the SNU21004 virus (parental virus) in three different culture systems, including Vero E6, DF-1, and ECEs. The summarized passage history is shown in Figure 1A. Briefly, the parental virus was serially passaged twelve times in Vero E6 cells. The passaged virus (V_12_) was later plaque-purified in monolayers of Vero E6 cells using 1% (*w*/*v*) SeaPlaque agarose (LONZA, Basel, Switzerland), as previously described [21]. Then, the virus clone with a dominant phenotype was randomly selected and further serially passaged into the DF-1 cells (V_12_D_x_) or ECEs (via the allantoic cavity route, V_12_E_x_) (X indicates the number of the passage), as previously described [22]. The presence of protein truncation or gene deletion of the G and/or SH gene in passaged viruses in vitro was identified using genome sequencing analysis. The genome sequences of the parental virus (SNU21004 strain) and its representative mutants were deposited in the GenBank database system. Cytopathogenic changes in aMPV-infected cells were also examined during the in vitro serial passage in this study. Mock-infected cells were included as the negative control. CPEs, such as syncytium formation, were observed daily during the serial passage process in both Vero E6 and DF-1 cells in this study. Cell viability was measured using a live/dead cell imaging kit (Invitrogen; #R37601) according to the manufacturer’s directions. For staining the cellular DNA, Hoechst 33342 Solution (Thermo Fisher, Waltham, MA, USA; #62249) was added at a concentration of 0.1% (*v*/*v*), and the images of the stained cells were visualized using the LSM 800 (ZEISS) confocal microscope set to a laser wavelength (FITC: 488 nm; TexRe: 561 nm; and H3342: 405 nm) and an excitation wavelength (FITC: 495 nm; TexRe: 592 nm; and H3342:348 nm) appropriate for the fluorescent labels used. The images were acquired using a C-apochromat 40×/1.2 oil objective. The pinhole was set to 0.98 Airy units, and the scanning speed was set at 9 μs per pixel. The laser power, gain, and offset settings were kept constant throughout the imaging of all samples to ensure comparability. The obtained images were processed and analyzed using the Zeiss LSM Zen software (version 1.8.23137.14).

### 2.3. Genome Sequencing and Mutational Pattern Analysis

To analyze complete genome sequences, viral RNA was extracted from either infected cells or samples of allantoic fluid, utilizing the viral gene-spinTM kit (iNtRON, Seongnam, Republic of Korea) in accordance with the guidelines provided by the manufacturer. The RT-PCR process was carried out with the use of EzAmp™ One-Step RT-PCR 5x Premix Kit (ELPIS-Biotech, Daejeon, Republic of Korea) adhering to the guidelines prescribed by the manufacturer. In this study, twelve primer sets were utilized to amplify overlapping genetic segments spanning the entire viral genome (Table S1). The RT-PCR conditions were RT process (50 °C for 10 min, followed by 95 °C for 3 min), 40 PCR cycles (95 °C for 20 s, 50 °C for 30 s, and 72°C for 2 min), and a final extension period at 72 °C for 5 min. The amplified PCR products were purified with the Expin™ Combo GP kit from (GeneAll^®^, Seoul, Republic of Korea) and subsequently sequenced using the ABI 3711 automatic sequencer (Macrogen Co., Seoul, Republic of Korea). The sequences of the overlapping fragments were combined into a single assembly using ChromasPro, version 1.5 (Technelysium Pty Ltd., Brisbane, Australia). The genome sequences of the variants were deposited in the GenBank database and assigned accession numbers (Table S2).

For the mutational pattern analysis of each of the viruses that had undergone serial passaging, genetic changes such as mutation, deletion, and insertion in the genome were examined through partial sequencing for each of the viral genes. The genome regions encompassing each of the genetic change sites were determined based on the results of the comparative genomic sequencing above. Each of the target genes was amplified using an EzAmp™ One-Step RT-PCR 5x premix kit (ELPIS-Biotech, Daejeon, Republic of Korea), and the resulting products were purified using an Expin™ Combo GP (GeneAll^®^, Seoul, Republic of Korea). Each of the PCR products above was cloned using RBC T&A cloning vector (RBC, Taiwan, China) and HIT competent cells (RBC, Taiwan, China) according to the manufacturer’s instructions. Ten colonies per PCR product were randomly selected and subjected to colony PCR using TaKaRa Ex Taq^®^ DNA polymerase (Takara, Shiga, Japan), and M13 primers and sequenced as described above. The frequency of genetic variation was analyzed as a percentage based on the 10 sequences for each site.

### 2.4. Assays for Virus Infectivity and Replication Kinetics

The titration of infectious viral particles was performed using an end-point dilution assay on Vero E6 cells, which were cultured in 96-well plates. Briefly, serial dilutions of the virus, ranging from 10^−1^ to 10^−7^, were prepared in DMEM. These dilutions were subsequently used to infect a monolayer of cells for a duration of one hour at 37 °C. The infected cells were rinsed twice with PBS, followed by the addition of fresh medium containing 2% FBS. The plates were incubated for 72 h with daily observations made to monitor CPE using an inverted microscope. Viral titers were determined and expressed as the 50% tissue culture infective dose (TCID50) per mL, calculated using the Reed and Muench method. The plates were incubated for 72 h with daily observations made to monitor CPE using an inverted microscope. Viral titers were determined as the 50% tissue culture infective dose (TCID50) per mL, calculated using the Reed and Muench method [23]. All samples in this study were tested in triplicate. The replication kinetics in vitro of the parental virus and its mutants were determined in Vero E6 or TOC. In brief, Vero E6 cells cultured in 24-well plates or TOCs cultured in 15 mL culture tubes were inoculated with 1 mL of virus stock solution (approximately 10^5^ TCID_50_/mL) for 1 h at 37 °C. Afterwards, the inoculum was removed from the cells or TOCs and reconstituted with 0.5 mL of culture medium supplemented with 2% (*v*/*v*) FBS. The infected cells or TOCs were incubated for 120 h at 37 °C, as described above. The samples were taken from the culture media of Vero E6 cells or TOCs at the time points 0, 24, 48, 72, 96, and 120 h post-inoculation. Viral RNAs from the culture media were extracted and amplified with primer for the qRT-PCR, as described above. The Ct values obtained from the samples at each time point were quantitatively analyzed by applying them to a previously established standard curve.

### 2.5. Transmission Electron Microscopy (TEM)

Virus particles in this study were examined via transmission electron microscopy. In brief, the virus stock solution was centrifuged at 65,000× *g* for 60 min at 4 °C in a T45 Beckman rotor. Concentrated virus pellets were re-suspended in phosphate-buffered saline (PBS) to approximately 1/100 of the original volume and then purified using discontinuous-sucrose-gradient 15%, 30%, and 50% (*w*/*v*) ultracentrifugation at 100,000 *g* for 2 h at 4 °C. Fractions containing viral particles were collected, and the virus particles were visualized via TEM using Talos L120C (FEI, Brno, Czech Republic) at 120 kV [24]. The virus particles were absorbed onto a carbon film 300 mesh-copper grid (EMS, CF300-Cu-UL) for 1 min and were negatively stained with 2% (*w*/*v*) uranyl acetate for 15 s. The excess stain on the grid was blotted on filter paper and air-dried. The morphology and sizes of the viral particles observed through the TEM were measured using an image J program (version 1.52) [25].

### 2.6. Animal Experiments

#### 2.6.1. Experiment 1: Chicken Experiments

Chicken experiments were conducted at the Preclinical Center of KBNP, Inc. (Yesan, Republic of Korea, approved as ID IACUC-KBNP C-23-01). A total of forty 6-week-old SPF chickens (Valo Biomedia, Adel, IA, USA) were used in this study, and they were composed of 4 groups (10 per group), comprising G1 (parental virus), G2 (mutant V_12_), G3 (mutant V_12_E_5_), and G4 (mock-infected). Groups G1 to G3 were inoculated with 10^4^ TCID_50_ per 0.1 mL through the intranasal route, and G4 was inoculated intranasally with PBS. Typical clinical signs and death were observed daily and recorded during the experiment. On the 5th day post-inoculation (PI), five chickens for each group were euthanized, respiratory tract samples including the turbinate, trachea, and lungs were taken, and the collected viral load in tissue samples was measured using quantitative RT-PCR assays. For cytokine profile analysis, whole blood was drawn in BD^®^ vacutainers with heparin sulfate on the 5th day PI, and peripheral blood mononuclear cells (PBMCs) were isolated. For serological tests, serum samples were collected from all remaining birds at two- and three-weeks PI.

#### 2.6.2. Experiment 2: Mouse Experiments

Mouse experiments were conducted at the Animal Center for Pharmaceutical Research (biosafety level 2) of Seoul National University (approved as IACUC-SNU-221228-1-1). A total of 8-week-old female BALB/c mice (*n* = 32) (KOATEC, Pyeongtaek, Republic of Korea) were used in this study. The mice were randomly separated into four groups (eight per group), comprising G1 (parental virus), G2 (mutant V_12_), G3 (mutant V_12_E_5_), and G4 (mock-infected) groups. Prior to inoculation, all the mice were anesthetized using Avertin (15 mg/kg, IP) (Virbac, Carros, France) and then infected intranasally with the challenge virus (1 × 10^4^ EID_50_/50 μL). Clinical signs and death were observed daily, and five mice per group were weighed daily for the first entire week and bled at 2 weeks. Three mice per group were euthanized 5 days post-inoculation, and lung samples were collected from all sacrificed mice. Lung samples were used for measurement of the viral load and early immune response against infection.

### 2.7. Cytokine and Early Immune Profile Analysis

For the analysis of cytokine gene expression, PBMCs were isolated from whole blood samples taken from infected chickens from Experiment 1 described above using the lymphoprep (Serumwerk, Bernburg, Germany) and the density gradient centrifugation method, as previously described [26]. Total RNAs were extracted from each of the PBMCs (10^5^ cells/mL) using the RNeasy micro kit (Qiagen). For cDNA synthesis of mRNA, Oligo-dt and Topscript Reverse transcriptase (Enzynomics, Daejeon, Republic of Korea) were used according to the manufacturer’s protocol. The cDNA synthesis was performed at 42 °C for 5 min, 50 °C for 1 h, and 95 °C for 5 min. Cytokine gene expression was determined at the mRNA level using the HS-qPCR (2× master mix, SYBR green, low ROX, ELPIS) and target cytokine primer (Table S3). Relative expressions of target genes were normalized to *GAPDH*, analyzed using the ΔΔCt method, and given as fold-changes calculated via mean ± SEM. Thermal cycling was performed in a CFX connect (BIO-RAD, Hercules, CA, USA).

In addition, the mRNA expression profile of innate immunity-related genes, including *Rig-*I, *Mda5*, *Lgp2*, *Mavs*, and *Actb*, was determined via qRT-PCR and an innate gene-specific primer (Table S3), as previously described [25]. Lung tissues collected from infected mice via experiment 2 above were used in this study. The ΔCt was calculated by subtracting the Ct value of the reference gene (*Actb*) from the Ct value of the target gene. The ΔΔCt was determined by measuring the difference in the ∆Ct values between infected lung samples and mock-infected lung samples. The fold change in gene expression for each experimental sample was calculated using the 2^−ΔΔCt^ formula. All samples were tested in triplicate in these experiments. For each primer pair, a melting curve was generated to ascertain the presence of a unique peak specific to the gene and to validate the absence of primer dimers.

### 2.8. Quantitative RT-PCR Assay

The replication kinetics of the parental virus and its mutants in vitro and in vivo were determined by quantifying the amount of RNA copy number using qRT-PCR and L protein primer. The cDNA was synthesized at 50 °C for 10 min and then heated at 95 °C for 3 min to activate the Taq polymerase. The PCR was performed with 40 repetitions at 98 °C for 15 s and at 60 °C for 40 s. SNU21004-V_5_ virus (5.8 TCID_50_/mL) was serially diluted to 10^−6^ for the determination of the standard curve, and CFX maestro 1.1 (Ver. 4.1.2433.1219) was used to convert the Ct values of each tissue sample to TCID_50_/mL using the following equations: Vero E6: y = −3.881x + 33.121, R^2^ = 0.0067; TOC: y = −4.16x + 34.982, R² = 0.9987.

### 2.9. Serological Tests

aMPV-specific antibodies induced via aMPV infection in the animal experiments above were quantified using both a commercial ELISA kit (ELISA; Bio-check, Fokkerstraat, The Netherlands) and a virus neutralization test (VNT). ELISA testing was performed in exact accordance with the manufacturer’s protocol. Serum samples with ELISA titers of >1656 were considered positive. The VNT was performed in 96-well plates with some modifications, as previously described [27]. Briefly, the two-fold dilution of heat-inactivated serum to be tested was mixed with an equal volume of SNU21004 virus (200 TCID_50_/0.1 mL). A total of 100 µL of the mixture was added to microplate wells (two wells per serum dilution) with a confluent monolayer of Vero E6 cells and incubated for 1 h. Then, the wells were washed twice with a sterile PBS solution. CPEs were recorded after a 7-day incubation at 37 °C. VNT titers were quantified as the reciprocal of the highest dilution of serum achieving a 50% reduction in CPE, calculated by employing the Reed and Muench method.

### 2.10. Statistical Analysis

Statistical analysis was conducted using IBM SPSS Statistics 26 (IBM SPSS Statistics for Windows, version 26.0., IBM Corp, Armonk, NY, USA). Differences between groups were assessed using an ANOVA with data that demonstrated a normal distribution. This was followed by Tukey’s honestly significant difference (HSD) test as a post hoc analysis. For non-parametric data, the Kruskal–Wallis test was applied, followed by a post hoc analysis using the Dunn–Bonferroni adjustment. A *p*-value < 0.05 was considered statistically significant.

### 2.11. Ethical Statement

This animal study’s protocol (for chickens and mice) was approved by the Institutional Review Board (or Ethics Committee) of the Preclinical Center of KBNP, Inc. (protocol code: IACUC-KBNP C-23-01; date of approval: 13 January 2023) and the Animal Center for Pharmaceutical Research (biosafety level 2) of Seoul National University (protocol code: IACUC-SNU-221228-1-1; date of approval: 1 May 2023).

## 3. Results

### 3.1. Serial Passage in Vitro of aMPV Field Strain SNU21004

The aMPV field strain SNU21004 was propagated and maintained in TOC from its first isolation from an affected chicken until its serial passage into other culture systems. The passage history of the SNU21004 virus in vitro is summarized in Figure 1. The SNU21004 virus was first passaged serially to adapt in Vero E6 cells. CPEs were apparently observed in five or more passages in Vero E6 cells. The viability of the Vero E6 cells was decreased following the infection of SNU21004-variants (Figure 1B, Table 1). After seven passages in the Vero E6 cells, the single virus clone was selected in a plaque assay and propagated in Vero E6 cells (called V_12_). Afterward, the cloned virus was further passaged in either DF-1 cells or ECEs (Figure 1A). At the first passage in DF-1 cells, the V_12_ virus developed CPEs, and these included focal degeneration such as pyknotic shrinking cells. The CPEs were stably observed in infected cells until the final (10th) passage in DF-1 cells (called V_12_D_10_). The infected DF-1 cells exhibited cell death due to virus replication. The infected DF-1 cells displayed a loss of membrane integrity, accompanied by a strong binding affinity of fluorescent red dye to DNA (Figure 1B, Table 1).

The V_12_ virus was also passaged serially in ECEs through the allantoic cavity (AC) route. No embryo mortality was observed during the 5-day incubation period until the first of the fourth passages in ECEs took place. Since the fifth passage in ECEs (called V_12_E_5_), some embryos of the inoculated eggs died during the incubation period, and the ECE-adapted viruses were detected at a low level (<10^4^ TCID_50_) from all inoculated eggs, regardless of embryo death.

### 3.2. Generation of aMPV Mutants via Serial Passage In Vitro

A parental virus (SNU21004) and two passage viruses, V_12_ (12 passages in Vero E6 cells) and V_12_E_5_ (12 passages in Vero E6 cells followed by 5 passages in ECEs), were subjected to full-length genomic sequencing. The nt and aa sequences of both viruses were compared with each other. As a result, the genome map for each of these viruses is represented in Figure 1C. The parental virus SNU21004 had a genome of 13,516 nt in size and exhibited no mutation in the genome during eight serial passages in TOC. However, genetic changes occurred during serial passage in Vero E6 and ECEs, as shown in Table 2. Surprisingly, the V_12_ virus had a single nucleotide (A) inserted into the poly-A region of the G gene (position 6098 of the genome), which resulted in +1 increase in the genome size (13,517 nt in size). This, in turn, resulted in −1 frameshifting at the aa position 28 of the G protein, which led to the creation of the stop codon at aa position 43 (S43*). The truncated G (ΔG mutant) with an N-terminal region (42 aa in length) only was 372 aa shorter than the full-length G (414 aa in length). In addition to G, mutations were identified at the eight aa positions in P (V92A and K104T), F (T83A), M2-2 (S71A), and L (M351K, I1061V, A1635T, and I1926V). The aMPV E_5_ virus had a short genome of 12,677 nt in size compared to the parental virus. The difference in genome length between the parental SNU21004 virus and V_12_E_5_ virus resulted from a deletion of 787 nt (positions nt 5748 to 6535) in the genomic region spanning from the SH gene (C-terminal) to the G gene (N-terminal). This, in turn, resulted in the C-terminal truncation of SH (ΔSH) and the entire truncation of the G gene (ΔG). In addition, the V_12_E_5_ virus had an additional aa substitution at two residues (I1803L, S1909G) in L alone compared to the V_12_ virus.

**Figure 2:**
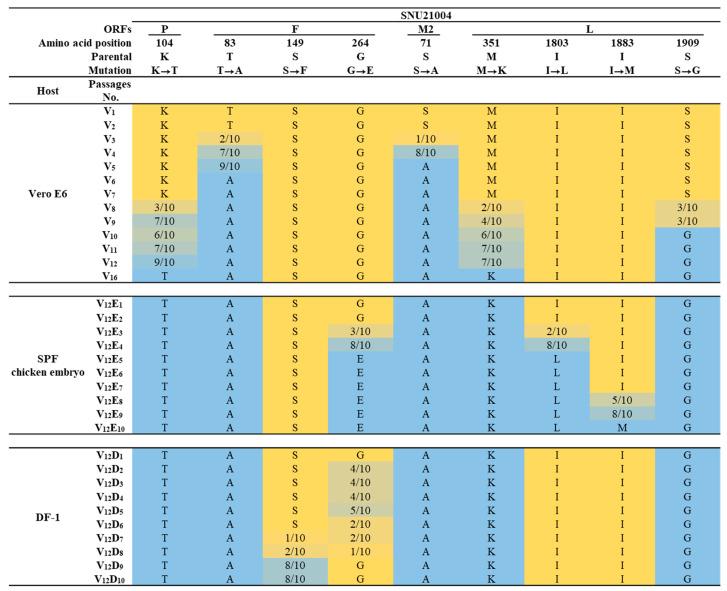
The frequency of gradual amino acid mutations per passage during the cellular adaptation of SNU21004. In the representation, the yellow color indicates the amino acid sequence originating from the SNU21004-WT, while blue designates the predominant amino acid sequence by mutation. The allele frequencies are denoted using a gradient of yellow to blue.

Thus, we further analyzed genetic changes in the genome for other remaining passage viruses by cloning the sequencing and investigating the patterns of the genetic change that might have occurred during adaptation in various viral culture systems. The sequence comparison results are shown in Figure 2. Genetic mutations dramatically occurred in SH and G proteins during adaptation in various viral culture systems. At least four distinct mutants with different-length truncations of G and/or SH were detected during serial passage in various viral culture systems: the mutants ΔG, ΔSH/G-1, ΔSH/G-2, and ΔSH/G-3 (Table 3). The mutants ΔG and ΔSH/G-1 were detected in the V_12_ virus and the V_12_E_5_ virus, respectively, via the genome sequencing analysis described above. The mutant ΔG first emerged after the fifth passage in Vero E6 cells and rapidly became predominant during the further serial passage in Vero E6 cells, which persisted even until the early passage in DF-1 and ECE. Meanwhile, the mutant ΔSH/G-1 emerged after the third passage in ECEs following the passage in Vero E6 cells, and then it rapidly became predominant during the further serial passaging in ECEs. The other two mutants (ΔSH/G-2 and ΔSH/G-3) emerged during the serial passaging in DF-1 cells. Like ΔSH/G-1, they also had various deletions in a region spanning from the SH gene (C-terminal) to the G gene (N-terminal); 688 nt (positions nt 5763 to 6451) for SH/G-2 and 1055 nt (positions 5641 to 6696) for ΔSH/G-3 (Figure 1C). The mutant ΔSH/G-2 first emerged in the fifth passage in DF-1 cells; then, the mutant was replaced by mutant ΔSH/G-3 with a broader gene deletion during the further serial passage.

Point mutation via a single a.a substitution also occurred at eight residues in other structural proteins. Serial passage in Vero cells resulted in point mutation at five residues in P (K104T), F (T83A and S71A), and L (M351K and S1909G). In particular, T83A (in F) and S71A (in M2) appeared from the third passage in Vero E6 and then dominated among virus populations since the fifth passage when CPE development was evident in infected Vero E6 cells. A later serial passage in DF-1 caused point mutations at two residues (S149F and G264E) in F, while the serial passage in ECEs resulted in point mutation at three residues in F (G264E) and L (I1803L and I1883M). Here, we selected four passage viruses V_12_, V_12_E_5_, V_12_D_5_, and V_12_D_10_ and used them in the current study as representative mutants ΔG, ΔSH/G-1, ΔSH/G-2, and ΔSH/G-3, respectively.

### 3.3. Morphological Comparison between the Parental Virus and the G-Truncated Mutant

**Figure 3:**
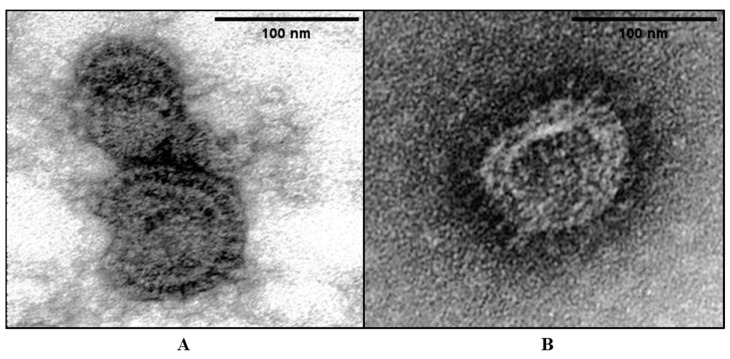
Ultrastructure of aMPV virions via negative staining. EM images of SNU21004-WT (**A**) and V_12_ (**B**). Two viruses were stained with 2% uranyl acetate. The virions were approximately 100–130 nm in diameter.

Electron microscopy was performed to investigate whether the G truncation affected the structure and shape of the viral particles. For this purpose, the parental virus (SNU21004) and G-truncated mutant ΔG were included. The morphology of viral particles was visualized via TEM, as shown in Figure 3. Uranium exhibits a strong reaction with phosphate and amino groups, commonly resulting in the staining of proteins, nucleic acids, and lipid membranes. The mutant ΔG viral particles resembled parental viral particles. The particles were 100 to 300 nm in diameter and had a spherical shape with an envelope. Glycoprotein projections were also observed on the envelope of both viruses. Comparisons between the parental and mutant ΔG virus revealed differences in the density of glycoprotein projection on the viral envelope. This observation implies that the reduced density of glycoprotein projection in the ΔG mutant virus might be attributed to the presence of F and SH proteins, along with the truncation of the C-terminal G.

### 3.4. Effect of G/SH Truncation on Virus Replication and Infectivity

In virus titration assays, the parental virus (SNU21004) had a titer of 10^5.5^ TCID_℃_ per mL in TOC, but it was not titrated in Vero E6 cells because there was not any CPE development before adaptation. Infectivity titers of four mutants, ΔG, ΔSH/G-1, ΔSH/G-2, and ΔSH/G-3, were 10^6.1^ TCID_50_/mL, 10^4.4^ TCID_50_/mL, 10^5.1^ TCID_50_/mL, and 10^5.2^ TCID_50_/mL, respectively, when measured in Vero E6 cells. This indicates that the infective titers of DF-1-adapted viruses (mutants ΔSH/G-2 and ΔSH/G-3) and ECE-adapted virus (mutant ΔSH/G-1) were approximately 10 to 100 times less than the mutant ΔG.

**Figure 4:**
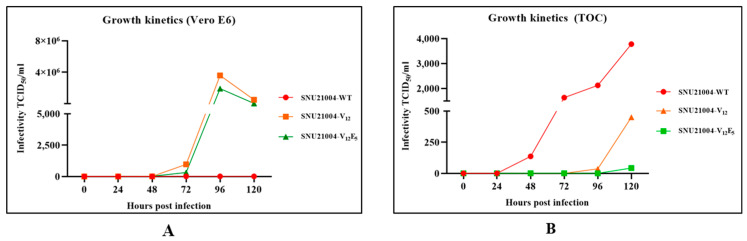
Viral replication of SNU21004-WT, V_12_, and V_12_E_5_ in Vero E6 or TOC. The cells and TOC were infected with a viral titer of 2 × 10^2^ TCID_50_ and incubated for a duration of 120 h. Samples were collected at designated time points for quantitative analysis. The Ct values obtained from these samples were then converted to TCID_50_/mL by applying them to a pre-established standard curve. The correlation coefficient (R^2^ value) for this standard curve was defined using the following formula (CFX maestro 1.1 (Ver. 4.1.2433.1219); Vero E6: y = −3.881x + 33.121, R^2^ = 0.0067; TOC: y = −4.16x + 34.982, R² = 0.9987).

To further analyze the effect of G and/or SH truncation on aMPV viral replication, time-course replication kinetics for two mutants, ΔG and ΔSH/G-1, were determined in Vero E6 cells and TOC via a quantitative RT-PCR assay, and the results were compared with that of the parental virus. As indicated in Figure 4, in Vero E6 cells, the amount of viral RNA of two mutants ΔG and ΔSH/G-1 in culture supernatant increased exponentially after 72 h post-infection and reached a peak. However, RNAs of the parental virus were undetectable for 120 h post-infection via quantitative RT-PCR, perhaps due to no adaptation taking place in Vero E6 cells. The TOC results were different from the results using Vero E6 cells (Figure 4). The parental virus replicated efficiently in TOC, while the two mutant viruses did not replicate. The amount of viral RNA in the parental virus in the TOC supernatant increased rapidly from 24 h post-infection until 120 h post-infection. The mutant ΔG virus was detected after 96 h post-infection, and the titer was significantly lower than that of the parental virus. The mutant ΔSH/G-1 virus was undetectable in the TOC supernatant until 120 h post-infection.

### 3.5. Effect of G/SH Truncation on Pathogenicity and Immunogenicity in Chickens

We then investigated whether G and/or SH truncation affected pathogenicity and immunogenicity in aMPV-infected chickens. For this purpose, SPF chickens were challenged with the parental virus SNU21004 (group G1), the mutant ΔG (group G2), and the mutant ΔSH/G-1 (group G3) through the intranasal route. A mock-infected control group (group G4) was also included. In group G1, mild clinical signs such as nasal discharge were observed in some of the SPF chickens (2/5) inoculated with the parental virus (Table 4). The clinical signs were apparent during the first week but were not detectable at later times. In groups G2 and G3, unlike in group G1, all the chickens exhibited no typical clinical signs during the experiment.

**Figure 5:**
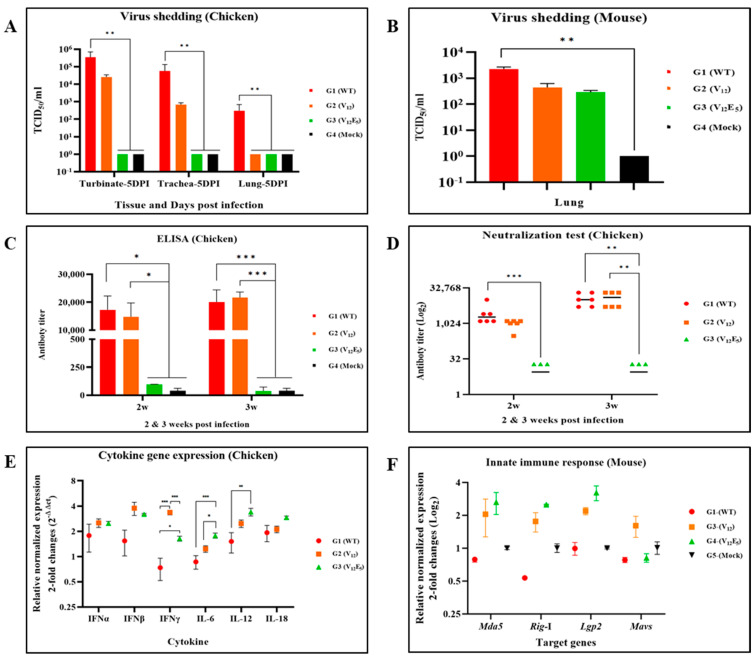
Evaluation of the pathological effects and immunogenicity of aMPV using an animal infection model. (**A**,**B**) The viral load was analyzed by quantifying the L RNA levels via RT-qPCR using RNA extracted from turbinate, trachea, and lung tissue. (**C**) ELISA antibody titer at 2 weeks post-infection (wpi) and 3 wpi. The mean titer of the antibody titer by group was calculated according to the manufacturer’s formula. A threshold value of 1,656 was established as the positive criterion, and this cutoff was delineated with a dotted line. (**D**) Viral neutralizing antibody titer at 2 wpi and 3 wpi. The normal serum of G4 (PBS-negative control) exhibited an antibody titer of 2^4.4^ against SNU21004-V_5_. The neutralizing antibody values for each group were normalized by subtracting the titer of the 2^4.4^ antibody. (**E**) Cytokine expression level of PBMC from infected chickens. The expression levels of the six cytokine genes were quantified and normalized using the 2^−ΔΔCt^ method. (**F**) Comparative analysis of mRNA expression level of genes associated with the innate immune response. The expression levels of the *Mda5, Rig-*I, *Lgp2*, and *Mavs* genes were quantified and normalized using the 2^−ΔΔCt^ method. There was no significant difference. The data shown are means ± SEM. The asterisks indicate the significant differences: *, *p* < 0.05; **, *p* < 0.01; and ***, *p* < 0.001.

Virus shedding was detected in respiratory samples (the nasal turbinate, trachea, and lung) in groups G1 and G2, while shedding viruses were not detectable in group G3. In group G1, the titers of virus shedding were higher in the nasal turbinate (10^5.5^ TCID_50_/mL), trachea (10^4.8^ TCID_50_/mL), and lung (10^2.4^ TCID_50_/mL), in that order. Such a tendency of viral titers in the nasal turbinate (10^4.4^ TCID_50_/mL), trachea (10^2.8^ TCID_50_/mL), and lung (undetectable) was also similarly observed in group G2, although the viral load of the G2 group was 10 to 100 times lower than that of the G1 group (Figure 5A, Table 4). Notably, in group G3, no virus was detectable in the respiratory tissue samples, even the nasal turbinate, during the first week after the viral challenge. The G4 birds showed no typical clinical signs or viral replication during the experiment.

To determine whether G/SH truncation affected the secretion of pro-inflammatory cytokines and interferons via viral infection in vivo, the cytokine expression in infected chickens was measured at the mRNA level by HS-qPCR using PBMC samples. Three interleukins (IL-6, IL-12, and IL-18) and three interferons (IFNα, IFNβ, and IFNγ) were tested. Two mutants, ΔG and ΔSH/G-1, upregulated the expression of pro-inflammatory cytokines and interferons compared to the parental virus (Figure 5E). Two mutants, ΔG and ΔSH/G-1, induced a remarkably high expression level of interferon-γ and interferon-β compared to the parental virus. In particular, the expression level of interferon-γ via the mutant ΔG was significantly higher than that of the mutant ΔSH/G-1_._ The expression level of Interleukin genes (IL-6, IL-12, and IL-18) was highest in chickens infected with the mutant ΔSH/G-1. The expression level of IL-6 and IL-12 induced via the mutant ΔG was higher than that of the parental virus.

We also evaluated humoral immunity induced via the parental virus and two mutants. Serum samples collected at two- and three-weeks PI were tested for aMPV-specific antibodies via ELISA and VNT (Figure 5C,D). As expected, the parental virus (group G1) induced a strong humoral response in infected chickens. The ELISA antibody titers were 17274 ± 2842.8 at 2 weeks PI and increased slightly at 3 weeks PI (ELISA titer 20050 ± 1431.1). The VN antibody titers were 3179 ± 1431.1 at 2 weeks PI and increased rapidly until 3 weeks PI (VNT titer 11926 ± 2855.8). The mutant ΔG (group G2) also induced a strong humoral immune response in infected chickens. The antibody titers (ELISA and VNT) of group G2 were not significantly different from those of the G1 birds. The mutant ΔSH/G-1 (group G3) induced an undetectable antibody response in chickens when measured using both ELISA and VNT.

### 3.6. Effect of G/SH Truncation on Pathogenicity and Immunogenicity in Mice

To determine whether G and/or SH truncation affected viral virulence and immunity in mammalian cells, BALB/c mice were challenged with the parental virus (group G1) and two mutant viruses, ΔG (group G2) and ΔSH/G-1 (group G3), via the intranasal route. A mock-infected control group (group G4) was also included.

BALB/c mice of all groups exhibited no clinical findings (e.g., loss in body weight, respiratory sign, or death) during the experiment (Figure 5B). aMPV-specific antibodies were also undetectable in all groups when measured 2 weeks post-inoculation via ELISA and VNT. Nevertheless, viral RNAs were detected in the lung tissues of the mice challenged with the parental virus and two mutants. The viral load in the mouse lungs of group G1 was higher than that of groups G2 and G3. Interestingly, the expression levels of innate immunity-related genes such as *Mda5, Rig-*I, and *Lgp2* induced via two mutant viruses, ΔG (group G2) and ΔSH/G-1 (group G3), were significantly higher compared to those induced via the parental virus, but the levels were low in all groups (Figure 5F).

## 4. Discussion

aMPV, like hMPV, is considered as a respiratory pathogen that mutates at a high frequency due to the nature of genetic instability of the RNA virus. aMPV replicates preferentially in the upper respiratory tract of poultry as a primary viral replication site (e.g., the nasal cavity and trachea) [19]. Thus, it has been reported that mutations such as deletion and truncation in some genes, especially SH and G, can arise from in vitro passaging the virus serially in a new host species such as Vero cells (the African green monkey kidney cell line) in vitro or from natural circulation in poultry in the field [16], perhaps as a mechanism of viral evolution for survival in a new host or environment. Inspired by the particular nature of aMPV, we attempted to generate genetically deleted mutant viruses by passaging the wild-type virus serially in various viral culture systems, including Vero E6 cells, DF-1 cells, and ECEs.

The aMPV strain SNU21004 used as the parental virus in this study originated from chickens. The parental virus was propagated and maintained in chicken TOC, showing active ciliary activity before serial passage in vitro in this study. The virus was maintained stably without any significant mutation during maintenance in TOC. However, as reported previously, genetic changes such as deletion and insertion in viral genes occurred when passaged serially in a viral culture system (Vero, DF-1, and ECEs) other than TOC in this study. In this study, at least four mutants, including ΔG, ΔSH/G-1, ΔSH/G-2, and ΔSH/G-3, were generated during serial passage in vitro. All these mutants had truncation of part or the entire region of the G protein. Mutants (ΔSH/G-1, ΔSH/G-2, and ΔSH/G-3) with complete truncation of the G protein also had truncation of the C-terminus of the SH protein. Nevertheless, in this study, these mutants did not have truncation with the same length and the same region of G or SH compared to previously reported mutants [18,28,29]. This suggests that, even if serial passages in vitro are carried out in a similar manner, the truncation region and degree of gene deletion may vary, depending on the passage environment, although changes in viral culture systems result in genetic mutations for viral evolution and adaptation. 

The G protein is known to play a role in viral attachment to cells, but it is not necessarily essential for viral infection, as reported in hMPAV and aMPV types A and C [17,18]. However, these reports have all been based on the results of replication in vitro from Vero E6 cells. Our findings were also supported by previous results suggesting that the G protein was not essential for viral infection and replication in Vero cells. However, in a viral infectivity test in vitro using chicken tracheal rings in this study, the G deletion (ΔG) significantly affected viral replication and production. In particular, a simultaneous truncation of G and SH resulted in the undetectable production of infectious virus in TOC. This suggests that the effect of G truncation on viral replication depends on the cultured cells used. 

In the in vivo experiments using chickens, the truncation of both G and SH (mutant ΔSH/G-1) resulted in viral shedding at an undetectable level, and the antibody response was also undetectable in infected chickens. Meanwhile, G truncation (ΔG) alone partly restricted viral replication and growth in the upper respiratory tract. These results were supported by results with viral replication kinetics obtained from chicken TOC. Similar restrictions in viral replication in vivo were also demonstrated in G-truncated hMPV studies [28,30] and in G-truncated aMPV type C studies [18,31]. In addition, the restricted viral replication of mutant ΔG in the upper respiratory tract resulted in viral attenuation in chickens. This partly appears to be due to impaired viral antagonism, an essential component of viral pathogenicity, since the involvement of G protein in viral antagonism has been demonstrated in hMPV [32]. 

Interestingly, the humoral immune response via mutant ΔG was strong enough to be comparable to that of the parental virus; however, the immune response to mutant ΔG in infected chickens was delayed compared to that of parental virus-infected chickens. To investigate the development of a strong humoral immune response in mutant ΔG-infected chickens despite restrictions in viral replication, we analyzed the mRNA expression of genes related to innate and adaptive immunity in mice and chickens during the early stages of viral infection. Notably, IFN**β** was significantly upregulated in ΔG mutant-infected chickens compared to those with parental virus. In addition, viral infection results in BALB/C mice revealed that mutant ΔG also upregulated the mRNA expression of cytosolic RNA helicases *Mda5* and *Rig-*I, which recognize double-stranded RNA motifs and uncapped 5′-triphosphates on viral RNA, respectively, and eventually trigger the production of IFNβ in epithelial cells [33]. Thus, the truncated G might contribute to the stimulation of IFNβ production via enhanced activation of innate immunity factors *Mda5* and *Rig-*I in respiratory epithelial cells, and the increased IFNβ expression level subsequently also affects restriction in viral replication in vivo. Therefore, our proposed mechanism suggests that the G-truncated mutant induced a stronger innate immune response than the parental virus due to impaired viral antagonism immediately after encountering the host immune system, which appears to have led to an increase in acquired immunity while mediating an enhancement in the viral clearance via adaptive immunity activation. If so, the increased innate and adaptive immunity in mutant ΔG-infected chickens may compensate for the restrictions in viral replication.

Notably, the mutant ΔG in our study induced high levels of neutralizing antibodies in infected chickens that were comparable to the parental virus. Given that the F protein is a major structural protein that induces protective humoral immunity such as neutralizing antibodies, the G truncation alone does not appear to affect the antigenic structure of the F protein. These characteristics of mutant ΔG in this study have important implications for the development of live attenuated vaccines. This is because this virus is attenuated in poultry, providing safety and, at the same time, triggering strong protective immunity. Importantly, if diagnostic tests targeting the G protein are used in combination with the mutant ΔG as a live attenuated vaccine, we can quickly differentiate between naturally infected and vaccinated birds. The potential of the mutant ΔG as a live attenuated vaccine candidate should be demonstrated through further study, including the genetic stability of the virus and the protective efficacy in chickens.

## Figures and Tables

**Table 1:** Evaluation of morphological alterations associated with cytopathic effects in three cell types infected by SNU21004-variants.

Cell	Group	Variant Characteristics	Cell Viability *
Vero E6	WT	-	Live cells ↑↑↑
	V_12_	P-K104T, G-A+, and L-M351K	Live cells ↑↑
	V_12_E_5_	F-G264E, ΔSH-G, and L-I1803L	Live cells ↑
	V_12_D_10_	F-S149F and ΔΔSH-G	Live cells
	Mock	-	Live cells ↑↑↑↑
DF1	WT	-	Dead cells ↑↑
	V_12_	P-K104T, G-A+, and L-M351K	Dead cells ↑
	V_12_E_5_	F-G264E, ΔSH-G, and L-I1803L	Dead cells
	V_12_D_10_	F-S149F and ΔΔSH-G	Dead cells ↑↑↑
	Mock	-	Live cells ↑↑↑↑

* The green channel represents live cells, while the red channel represents dead cells. (↑) This grading ranges from 0 to 4, where number of arrow represents a level of live/dead cell.

**Table 2:** Comparison of nucleotide and amino acid sequences of WT with V_12_, and V_12_E_5._

Gene	Length of 21004-WT/V_12_/V_12_E_5_ (a.a)	SNU21004		
		WT	V_12_^a^	V_12_E_5_^b^
			Missense Mutation Compared to WT	
G+C (%)	-	43.2	42.23	43.36
Full (nucleotide)	-	13,516	13,517	12,677
3’-UTR	1–55	-	A32T	-
N	56–1231 (391)	-	-	-
P	1255–2094 (279)	-	V92A and K104T	V92A and K104T
M	2120–2884 (254)	-	-	-
F	2945–4566 (538)	-	T83A	T83A and G264E
M2-1	4589–5148 (186)	-	-	-
M2-2	5106–5327 (74)	-	S71A	S71A
SH	5380–5907 (175)	-	-	Truncated a.a (123–175)
G	6016–7260 (414)	-	Truncated a.a (43–414)	Truncated a.a (1–414)
L	7380–13,394 (2004)	-	M351K, I1061V, A1637T, and I1926V	M351K, I1061V, I1803L, and S1909G
5’-UTR	13,395–13,536	-	-	-

^a^ In V_12_, nucleotide A was inserted at position 6098 of the genome. ^b^ V_12_E_5_ has a nucleotide deletion at position 5748-6534 of the genome.

**Table 3:** Truncation of G protein via nucleotide insertion/deletion during cell passage.

Mutant	Gene	Cell	INDELs (Insertion/Deletion)	Passage Range
ΔG	*G*	Vero E6	A+: A insertion (nt6098)	V_6_–V_16_
ΔSH/G-1	*SH-G*	ECE	5748 to 6534	V_12_E_3_–V_12_E_10_
ΔSH/G-2	*SH-G*	DF-1	5763 to 6451	V_12_D_5_–V_12_D_8_
ΔSH/G-3	*SH-G*	DF-1	5641 to 6696	V_12_D_8_–V_12_D_10_

**Table 4:** Results of the viral load measurement in the respiratory tract of infected chickens after the APV challenge by group.

Group	Strains of SNU21004	Infection Dose/0.1 mL (TCID_50_/Chicken)	Clinical Sign (%)	Viral Load					
				Turbinate		Trachea		Lung	
			Nasal Discharge	Positive Rate *	Mean Titer	Positive Rate	Mean Titer	Positive Rate	Mean Titer
G1	WT	4.0	40%	5/5	5.5	5/5	4.8	5/5	2.4
G2	V_12_	4.0	0%	5/5	4.4	3/5	2.8	0/5	0.0
G3	V_12_E_5_	4.0	0%	0/5	0	0/5	0	0/5	0.0
G4	Mock	-	0%	0/5	0	0/5	0	0/5	0.0

* The number of positive detections from five chickens.

## Data Availability

The whole genome sequences of strains were deposited on GenBank under the following accession numbers: SNU21004-WT, OM249786; SNU21004-V_12_, OR461284; and SNU21004-V_12_E_5_, OR461285. The data are available after 15 September 2024.

## Supplementary Materials

The following supporting information can be downloaded at: https://www.mdpi.com/article/10.3390/vaccines12010106/s1, Table S1: primer set for disease diagnosis; Table S2: accession number registered in GenBank; Table S3: primer set for the detection of innate immune-related genes; and Figure S1: during multi-passage, additional deletion of the SH-G occurred in the SNU21004-V12Dx virus.
