# Supplementary material for: Effects of G and SH Truncation on the Replication, Virulence, and Immunogenicity of Avian Metapneumovirus
Source: Vaccines (Basel). 2024 Jan 21;12(1):106. doi: 10.3390/vaccines12010106 (PMC10818707; doi:10.3390/vaccines12010106)
Supplement: Supplementary file 1 [file vaccines-12-00106-s001.zip › vaccines-2799158-supplementary.pdf]

Table S1. Primer set for disease diagnosis.

No.	Target gene (Protein)	Primer	Forward (5' to 3')	Reverse (5' to 3')	Amplicon (bp)	Usage
1	L	aMPV-L	GTTGCCTTGCATGCCATTG	GCCTGTCAATTGGGTCTTCTAC	210	Viral RNA detection (RT, qRT-PCR)
2	N	FR1	ACGAGAAAAAAACGCATTCAAGTCAC	GCATTCCCCAAAACAACAC	1032	Full genome sequencing, Gene cloning
3	P	FR2	CATCTTCATGCAAGCTTATGGAG	GGCACACCTTGATAGGTATC	1374	
4	M	FR3	CAAAAATAGGAGATGGGAGTGTG	GGTTACAGTGCTGCATGATTC	1107	
5	F	FR4	CTCGGTGCAGGAACACAAG	CTCCAAATATTGGGAGTTGC	1012	
6	F, M2-1	FR5	CAGTGCAGGAATAACTTCC	GTCTCGTGCAGATTTCACATCA	1363	
7	M2-2. SH	FR6	GACCATGTATTGTTAGTGCG	GTTCTTGGTGCACATACAGTG	1196	
8	G	FR7	AAACTCTGATGGATGCCTAG	TTATTGACTAGTACAGCACCAC	1510	
9	L	FR8	CAGCAAATCAACTCCTCCAG	GTACCACTTAACAACTCATC	1575	
10	L	FR9	CATCATTTGGCTGCATCTTG	CATGCCTATAAGTGCATATC	1500	
11	L	FR10	CTATCTTCAGTGAAAGCTAG	CTATTGTTGTGACGCAATTC	1526	
12	L	FR11	GTTATGCAAGATGTAGGGCCTG	GATTGTGGTGTCATACAGCATC	1446	
13	L	FR12	CATGGTTCCTGATGAGCTTC	ACGGCAAAAAAACCGTATTCAATACA	1803	

Table S2. Accession number registered in Genbank.

aMPV strain	Accession number
SNU21004WT	OM249786
SNU21004-V_{12}	OR461284
SNU21004-$V_{12}E_5$	OR461285

Table S3. Primer set for the detection of innate immune-related genes.

Target gene		Sequence (5' -> 3' direction)
IFN-α	F	ATCCTCCAGCACCTCTTCAA
	R	AATCGTTGTCGTGGAGGAAG
IFN-β	F	CCTCCAACACCTCTTCAACATG
	R	TGGCGTGTGCGGTCAAT
IFN-γ	F	GTGAAGAAGGTGAAAGATATCATGGA
	R	GCTTTGCGCTGGATTCTCA
IL-6	F	GCTCGCCGGCTTCGA
	R	GGTAGGTCTGAAAGGCGAACAG
IL-12	F	TGGTCCACGCTTTGCAGAT
	R	AAGGTTAAGGCGTGGCTTCTTA
IL-18	F	ACGTGGCAGCTTTTGAAGAT
	R	GCGGTGGTTTTGTAACAGTG
GAPDH	F	CCCCAATGTCTCTGTTGTTGAC
	R	CAGCCTTCACTACCCTCTTGAT
Mouse *Actb*	F	ATT GGCAACGAGCGGTTCC
	R	AGCACTGTGTTG GCATAGAGG
Mouse *Rig-i*	F	GAGAGTCACGGGACCCACT
	R	CGGTCTTAGCATCTCCAACG
Mouse *Mda5*	F	TGATGCACTATTCCAAGAACTAACA
	R	TCTGTGAGACGAGTTAGCCAAG
Mouse *Lgp2*	F	CAGCCTAGTCTGCTGCTATTC
	R	CCAGAGCAGGTAAGATCACTT
Mouse *Mavs*	F	CTGGCTGATCAAGTGACTCG
	R	AATGCAGAGGGTCCAGAAAC

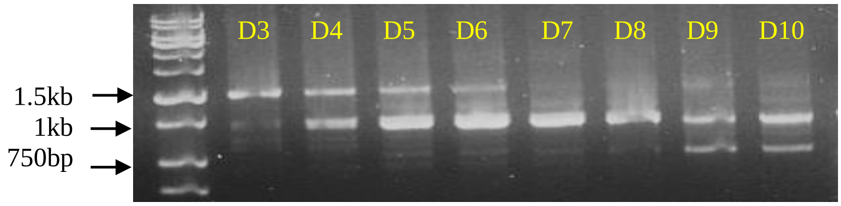

Figure S1. During multi-passage, additional deletion of the SH-G occurred in the SNU21004-$V_{12}D_x$ virus.
